# Erythrocyte-Derived Microparticles Supporting Activated Protein C-Mediated Regulation of Blood Coagulation

**DOI:** 10.1371/journal.pone.0104200

**Published:** 2014-08-19

**Authors:** Ruzica Livaja Koshiar, Sofia Somajo, Eva Norström, Björn Dahlbäck

**Affiliations:** Department of Laboratory Medicine, Division of Clinical Chemistry, Lund University, Skåne University Hospital, Malmö, Sweden; National Cerebral and Cardiovascular Center, Japan

## Abstract

Elevated levels of erythrocyte-derived microparticles are present in the circulation in medical conditions affecting the red blood cells. Erythrocyte-derived microparticles expose phosphatidylserine thus providing a suitable surface for procoagulant reactions leading to thrombin formation via the tenase and prothrombinase complexes. Patients with elevated levels of circulating erythrocyte-derived microparticles have increased thrombin generation in vivo. The aim of the present study was to investigate whether erythrocyte-derived microparticles are able to support the anticoagulant reactions of the protein C system. Erythrocyte-derived microparticles were isolated using ultracentrifugation after incubation of freshly prepared erythrocytes with the ionophore A23187 or from outdated erythrocyte concentrates, the different microparticles preparations yielding similar results. According to flow cytometry analysis, the microparticles exposed phoshatidylserine and bound lactadherin, annexin V, and protein S, which is a cofactor to activated protein C. The microparticles were able to assemble the tenase and prothrombinase complexes and to stimulate the formation of thrombin in plasma-based thrombin generation assay both in presence and absence of added tissue factor. The addition of activated protein C in the thrombin generation assay inhibited thrombin generation in a dose-dependent fashion. The anticoagulant effect of activated protein C in the thrombin generation assay was inhibited by a monoclonal antibody that prevents binding of protein S to microparticles and also attenuated by anti-TFPI antibodies. In the presence of erythrocyte-derived microparticles, activated protein C inhibited tenase and prothrombinase by degrading the cofactors FVIIIa and FVa, respectively. Protein S stimulated the Arg306-cleavage in FVa, whereas efficient inhibition of FVIIIa depended on the synergistic cofactor activity of protein S and FV. In summary, the erythrocyte-derived microparticle surface is suitable for the anticoagulant reactions of the protein C system, which may be important to balance the initiation and propagation of coagulation in vivo.

## Introduction

Microparticles (MPs) are defined as membrane-derived vesicles smaller than 1 µm that are shed from any cell type in response to cell activation, cell stress or apoptosis [1]–[3]. The cellular origin of the MPs can be identified by the presence of surface molecules from their parent cells. In blood circulation, MPs originating from platelets, erythrocytes, leukocytes, and endothelial cells can be identified [2]. The most abundant MPs arise from platelets [3]–[5], followed by MPs from endothelial cells, granulocytes and erythrocytes (eryMPs) [4].

Apart from bearing the surface molecules of their mother cell, another hallmark of many MPs is the exposure of negatively charged phospholipids (phosphatidylserine) in the outer cell membrane. Indeed, eryMPs isolated from blood units were shown to stain positively for phosphatidylserine [6], as do eryMPs isolated from patients [7]. Phosphatidylserine positive MPs have previously been shown to provide suitable surface for the assembly and consequent activation of coagulation factors [8]–[12]. Upon initiation of coagulation, a series of enzyme activations takes place on the negatively charged surface. Two key reactions are the activations of coagulation factor X (FX) and prothrombin. The Xase complex comprising the enzyme FIXa and its cofactor FVIIIa, activates FX, whereas the prothrombinase (PTase) complex (FXa plus its cofactor FVa) activates prothrombin. The anticoagulant protein C system tightly regulates these reactions [13]. Activated protein C (APC), together with its cofactor protein S, targets and degrades FVa and FVIIIa resulting in inhibition of the coagulation pathway.

Increased concentrations of circulating eryMPs have been found in patients with diseases affecting the red blood cells, such as sickle cell anemia, paroxysmal nocturnal hemoglobinanemia (PNH) and β-thalassemia [14]–[16]. Presence of eryMPs is specifically correlated to in vivo markers of increased coagulation [16] and several studies have shown that eryMPs have the ability to support blood coagulation in vitro [6], [17]. However, there are few studies of the anticoagulant APC-system in relation to eryMPs. It has been shown that irreversibly sickled red blood cells and eryMPs can bind protein S [18] and that the red blood cells from sickle cell disease patients support APC-mediated degradation FVa [19]. In addition, platelet-derived MPs were recently shown to stimulate APC-mediated regulation of coagulation in a protein S dependent manner through degradation of both FVa and FVIIIa [20].

In this study eryMPs were investigated for their ability to bind protein S and support the APC-system in regulation of the Xase and PTase reactions. Both cofactors FVa and FVIIIa were inhibited by APC and protein S on the surface of eryMPs. In plasma-based thrombin generation assays, the pro-coagulant effect of eryMPs was suppressed by addition of APC. The anticoagulant effect of APC in plasma depended upon the presence of protein S.

## Materials and Methods

### Reagents

Anti-CD235a-PE used in flow cytometry was from BD Biosciences, Franklin Lakes, NJ, USA (isotype control mouse IgG2-PE). Rabbit-anti-protein S (A0384 DAKO, Glostrup, Denmark) was labeled with Alexa488 using the Microscale Protein Labeling kit (A30006, Life Technologies, Invitrogen, Carlsbad, CA, USA), following the manufacturer's instructions. Buffer exchanges in the labeling process were performed with ZEBA desalting spin columns (Thermo Fisher Scientific Inc. (Pierce), Rockford, IL, USA) and concentrations of antibodies were determined using NanoDrop 2000 (Thermo Fisher Scientific Inc.). Calibration beads (Flow Count) were purchased from Beckman Coulter. FITC-labeled lactadherin was from Haematologic Technologies Inc., Essex Junction, VT, USA. Protein S used in flow cytometry was purified from human plasma as described [21]. Protein S used in coagulation assays was purchased from Enzyme Research Laboratories (ERL, South Bend, IN, USA). Ionophore A23187 (calcimycin) was from Life Technologies, Invitrogen. Human FXa was from ERL, FVIII was from Octapharma (Lachen, Switzerland) and human FIXa and corn trypsin inhibitor (CTI) were from Haematologic Technologies Inc. Human FV was purified from plasma as described [22], with minor modifications [9]. Bovine FX [23], and human prothrombin [24] were purified from plasma as described. Human APC was obtained from recombinant protein C expressed, purified and activated, as described [25]. Human α-thrombin was prepared from purified prothrombin, as described [26]. Hirudin was purchased from Pentapharm, Basel, Switzerland. Collagen (native fibrils type 1 form equine tendons) was from Chrono-Log Corp. (Haverton, PA, USA). Bovine serum albumin (BSA) was from Sigma-Aldrich (St Louis, MO, USA). The natural phospholipids phosphatidylserine (brain extract) and phosphatidylcholine (egg extract) were from Avanti Polar Lipids Inc. (Alabama, USA). Ready gels (4–15% TGX) and stacks for SDS-PAGE and western blotting were from (Bio-Rad, Hercules, CA, USA). Outdated erythrocyte concentrate was kindly provided by the blood bank at the hospital in Växjö, Sweden.

### Ethics statement

Venous blood was collected from healthy volunteers after informed written consent (approved by the Regional Ethical Review Board, Lund, Sweden, permission number 2012/202). The samples were anonymized, and stored no longer than one month.

### Preparation of erythrocyte-derived microparticles

EryMPs generated by the calcium ionophore A23187 were prepared essentially as described before [17]. Venous blood was drawn (Vacutainer Citrate 4.5 mL tubes, BD, Franklin Lakes, NJ, USA) and supplemented with CTI (50 µg/mL) to avoid activation of the contact phase. Erythrocytes were pelleted by centrifugation (250 g, 15 min). Supernatant and buffy coat were removed and the pelleted erythrocytes were pooled and centrifuged as above. The erythrocytes were washed 3 times with double volume HN-buffer (25 mM Hepes (4-(2-Hydroxyethyl-1)-1-piperazineethanesulfonic acid), 150 mM NaCl, pH 7.5), (centrifugation 2 500 g, 5 min) followed by resuspension in equal volume of HN-buffer and activation by addition of CaCl_2_ (3 mM) and calcium ionophore A23187 (final concentration (FC): 10 µM) (shake 170 rpm, 37°C, 1 h). Residual erythrocytes were removed by centrifugation (2 500 g, 15 min) and the supernatant was ultra centrifuged (100 000 g, 4°C, over night). The eryMP pellet was resuspended in 3 mL HN-buffer and supplemented with 0.5% BSA (HNBSA) (aliquots stored at −80°C). The concentration of eryMPs was determined by flow cytometry. The MPs present in the outdated erythrocyte concentrate were prepared by the method of Rubin et al [27].

### Flow cytometry

EryMPs were analyzed in flow cytometer FC500 (Beckman Coulter, Brea, CA, USA), using IsoFlow Sheath Fluid (Beckman Coulter) as fluid phase. The laser was set at 488 nm, thresholds for forward scatter (FS) and side scatter (SS) were set to 2. Fluorescence channels, FS and SS were set at logarithmic gain. The flow cytometry data were analyzed using FlowJo 8.7.1 (Tree Star, Inc., Ashland, OR, USA). Data was collected with live gate on erythrocyte surface marker glycophorin A (anti-CD235a-PE detected in FL2).

#### Lactadherin binding

Phosphatidylserine exposure was analyzed by binding of FITC-labeled lactadherin [28]. EryMPs (10×10^6^/mL) were labeled with anti-CD235a-PE (2.5 µg/mL) and lactadherin-FITC (0–160 nM) in a total volume of 50 µL HNBSA, 2 mM CaCl_2_ (15 min, RT) followed by dilution to 500 µL (HNBSA, 2 mM CaCl_2_). EryMPs were collected (>10 000 CD235a-positive events) and exposure of phosphatidylserine was detected in FL1 by the binding of lactadherin-FITC. The outdated eryMP were also tested for annexin V binding (see supplement).

#### Protein S binding

Binding of added protein S to the eryMPs was analyzed after a two-step labeling procedure. MPs (10×10^6^/mL) were pre-incubated with protein S (0–62.5 µg/mL) and anti CD235a (2.5 µg/mL) in a total volume of 40 µL for 15 min, RT. Anti protein S-Alexa Fluor 488 (10 µL, 0.7 mg/mL) was added and the labeling mix was further incubated 15 min followed by dilution to 500 µL with HNBSA, 2 mM CaCl_2_. In negative binding control, the labeling procedure was repeated but using IsoFlow with 5 mM EDTA (ethylenediaminetetraacetic acid) as diluent. A minimum of 10 000 CD235a-positive (FL2) events were collected and protein S binding was detected in FL1.

#### Counting

To determine the MP concentration, 5 µL eryMPs were labeled with 2.5 µg/mL anti-CD235A. Calibration beads (50 µL) were added just before data collection. CD235a-positive events (FL2) were collected (live gate, >10 000 events) and the concentration of eryMPs was determined by comparison to the predefined bead concentration.

### Xase assay

EryMPs were titrated in Xase assay to evaluate their ability to support formation of FXa. A mix containing FVIII (1 U/mL) and FIXa (8.9 nM) was treated with 0.1 U/mL thrombin (37°C, 3 minutes) to activate FVIII. The reaction was stopped with 0.3 U/mL hirudin. The activation mix was diluted with FIXa (8.9 nM) to obtain 370 mU/mL FVIIIa. To 60 µL activation mix 40 µL eryMPs (FC: 0–16×10^6^/mL) and bovine FX (FC: 0.5 µM) were added and incubated for 3 minutes. The reaction was stopped by dilution 1/16 in ice cold EDTA stop buffer (50 mM Tris (tris(hydroxymethyl)aminomethane)-HCl, 100 mM NaCl, 20 mM EDTA 1% polyethylene glycol 6000 (PEG6000), pH 7.9). Formed FXa was assessed kinetically by conversion of the chromogenic substrate S-2765 (Chromogenix, Milan, Italy). Data were collected every 30 s for 15 minutes in Infinite 200 microplate reader (Tecan, Männedorf, Switzerland). Known concentrations of FXa were used to obtain a calibration curve.

### FVIIIa degradation assay

To evaluate the APC mediated inactivation of FVIIIa, FVIII was activated as above and a degradation mix consisting of 210 mU/mL FVIIIa, 5 nM FIXa, 16×10^6^/mL eryMPs, 0–5 nM APC, with or without FV (2 nM) and/or protein S (33 nM) in a total volume of 105 µL was prepared. After incubation (2.5 min 37°C), 20 µL bFX (FC: 0.5 µM) was added and remaining FVIIIa activity was calculated from measurement of formed FXa (determined as above).

### Prothrombinase assay

The ability of eryMPs to support formation of thrombin in the PTase reaction was measured as described [29]. The final concentrations in the assay were 0.5 µM prothrombin, 5 nM hFXa, 40 pM FVa, 3 mM CaCl_2_ and 0–6×10^6^ eryMPs/mL. The thrombin formed was measured kinetically with chromogenic substrate (S-2238, Chromogenix) in Infinite 200 microplate reader (Tecan). Data were collected every 30 s for 15 minutes. Human thrombin of known concentrations were used to obtain a calibration curve for the conversion of the substrate.

### FVa-degradation assay

Plasma purified FV (5.5 nM) was activated with 1 U/mL of thrombin 10 min, 37°C, and the reaction was stopped by addition of 2 U/mL hirudin. A degradation mix (50 µL), containing eryMPs (6×10^6^ MPs/mL), APC (0–2 nM), FVa (0.8 nM) in absence or presence of 100 nM protein S was incubated 10 min at 37°C to allow APC-mediated degradation of FVa. To study degradation over time, aliquots were removed at different time points after addition of APC (FC: 60 pM). To stop the reaction, the mix was diluted 1/10 in ice cold HNBSA, 5 mM CaCl_2_. To evaluate the remaining FVa-activity, a PTase assay was performed as described above. Extruded phospholipid vesicles (phosphatidylserine/phosphatidylcholine 10/90) were included in the PTase assay, as insufficient concentrations of membrane surface were provided by the MPs. The final concentrations in the PTase assay were 0.12×10^6^/mL of eryMPs, 50 µM phospholipids, 10 nM FXa, 0.5 µM prothrombin and 16 pM FVa/FVi.

### Electrophoresis and immunoblotting

APC-mediated degradation of monoclonal antibody-affinity purified FVa was analyzed using SDS-PAGE and western blotting as described [30]. FVa-degradation was performed as above, however aliquots removed at different time points diluted in an equal volume of denaturing, reducing sample buffer. Samples were applied to, and separated in a 4–15% ready gel. Separated proteins were transferred to a PVDF-membrane using pre-packed transfer stacks (Trans-Blot Turbo Bio-Rad), in a Trans Blot Turbo device (Bio-Rad) (30 minutes, 1A, 25V). The FV-fragments were identified using a monoclonal antibody (AHV 5146, Haematologic Technologies Inc. Essex Junction, VT, USA (epitope between amino acids 307 and 506)) followed by horseradish peroxidase conjugated goat-anti-mouse antibody (DAKO). Membranes were developed using enhanced chemiluminescence substrate in Chemdoc XRS (Bio-Rad) CCD-camera.

### Analysis of kinetic data

FVa is cleaved by APC at Arg506 and Arg306 by the two pathways presented below (Eq. 1, and Eq. 2), resulting in the rate constants k_506_, k_306_ and k′_306_ (FVa_int_ = FVa cleaved at Arg506, with intermediate activity, FVa*i* = completely inactivated FVa) [31].
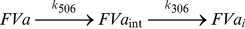
(1)

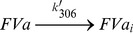
(2)


To determine the rate constants for FVa-degradation on the MPs, the kinetic data was fitted to the modified pseudo first-order equation (Eq. 3).
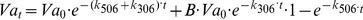
(3)


The equation was modified based on the reported similar rates of k_306_ and k′_306_ [31]. Va_t_ represents total FVa activity, Va_0_ is the FVa activity at time (t) 0 and B is the remaining activity when FVa is cleaved at only Arg506.

### Thrombin generation assay

Citrated whole blood was collected from healthy volunteers after informed consent and supplemented with 50 µg/mL CTI to inhibit contact phase activation. Platelet poor plasma was prepared by double centrifugation (2 000 g, 15 min) and frozen in aliquots. Supernatant from thawed, centrifuged (20 000 g, 45 min) plasma (40 µL) was diluted with 40 µL HNBSA in absence or presence of antibodies against protein C (polyclonal, DAKO), protein S (in house MK21 (monoclonal) against the Gla domain [32]) or TFPI (sheep polyclonal PAHTFPI-S from Haematologic Technologies) (FC 100 µg/mL) (incubated 10 min, 37°C). Preparations of eryMPs (0–28×10^6^/mL) in HNBSA, were pre-incubated with 3 pM tissue factor ((TF) (Dade Innovin, Siemens, Erlangen, Germany)) and APC (0–86 nM) for 1 min at 37°C. EryMPs/TF/APC (30 µL) was added to the plasma/antibody mix (total volume 110 µL) and the reaction was started by addition of 20 µL fluorogenic thrombin substrate (2 mM Z-Gly-Gly-Arg-AMC, Bachem, Bubendorf, Switzerland) containing 108 mM CaCl_2_. The final concentrations were 0.7 pM TF, 300 µM substrate, 16.7 mM CaCl_2_, 0–20 nM APC and 0–3×10^6^ eryMPs/mL. All reagents were diluted in HNBSA. The formation of thrombin was monitored with accumulated fluorescence in Infinite 200 (Tecan) microplate reader at excitation wavelength 360 nm and emission wavelength 460 nm. Data were collected every 20 s for 1 hr. at 37°C. The output data were analyzed using GraphPad Prism 5.0 (GraphPad Software Inc., La Jolla, CA, USA.) and the accumulative fluorescence was transformed to substrate conversion rate by derivatizing the obtained data.

## Results

### Characterization of erythrocyte-derived microparticles

**Figure 1:**
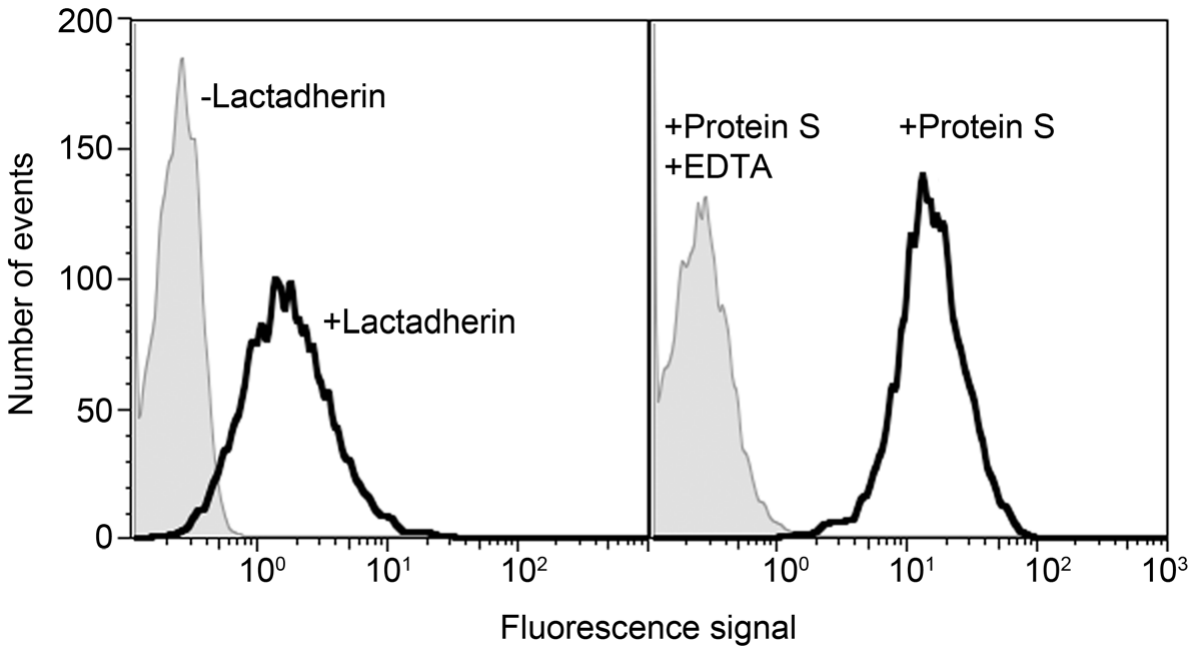
Flow cytometry analysis of erythrocyte-derived MPs – exposure of phosphatidylserine and binding of protein S. EryMPs (10×10^6^/mL) were labeled with anti-CD235A-PE (2.5 µg/mL) and in the left panel lactadherin-FITC (160 nM) (total volume 50 µL) and analyzed in the FC500 flow cytometer. CD235A-positive events were collected and plotted against the fluorescence (log) signal in FL1 (lactadherin-FITC) to identify phosphatidylserine (shaded histogram: negative control where lactadherin was excluded). In the right panel protein S (62.5 µg/mL) was incubated with eryMPs (10×10^6^/mL) and anti-CD235A-PE (2.5 µg/mL). After incubation (15 min) 10 µL, 0.7 mg/mL alexa488-conjugated polyclonal antibody against protein S was added (final volume 50 µL) and incubated for 15 min and analyzed. CD235A-positive events were collected and plotted against fluorescence (log) signal in FL1 (anti-protein S-Alexa488). Labeling process was performed in presence 2 mM CaCl_2_ or 5 mM EDTA (negative control, shaded histogram).

MPs were either isolated from erythrocytes after treatment with the calcium ionophore A23187 or from an outdated erythrocyte concentrate using ultracentrifugation. The MPs isolated from the two sources yielded qualitatively similar results. The presented experiments are from the A23187-induced MPs and those using outdated MPs are shown in the supplement. The MPs were analyzed by flow cytometry (Figure 1). CD235a was used as erythrocyte marker to define the MPs. The MPs bound lactadherin, demonstrating phosphatidylserine exposure on their outer surface. They also bound protein S in a dose-dependent manner. The protein S binding was calcium-dependent and addition of EDTA totally abolished the binding.

### Pro- and anticoagulant reactions supported by erythrocyte microparticles

**Figure 2:**
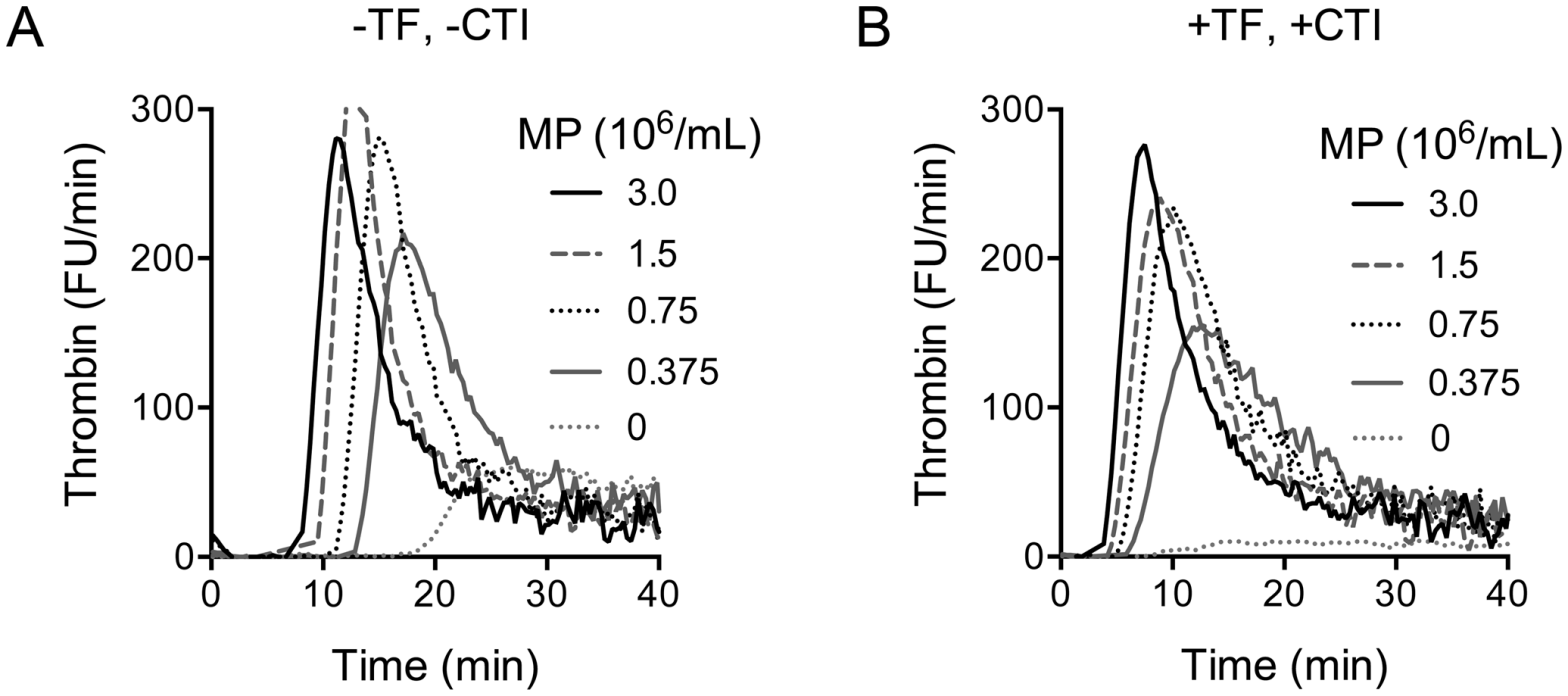
Erythrocyte-derived microparticles supporting extrinsic and intrinsic pathways. Platelet poor plasma with or without CTI (50 µg/mL) was diluted ½ and 80 µL was added to a mix of eryMPs and TF (30 µL). Thrombin generation was initiated by addition of 20 µL CaCl_2_ solution containing the thrombin substrate Z-Gly-Gly-Arg-AMC. The accumulated fluorescence was monitored and presented as the first derivative representing thrombin activity. Concentrations in the assay were: 0–3×10^6^ eryMPs/mL, 0 or 0.7 pM TF, 300 µM Z-Gly-Gly-Arg-AMC, 16.7 mM CaCl_2_. A) Thrombin generation initiated by intrinsic pathway (no added TF or CTI). B) Thrombin generation initiated via extrinsic pathway with TF (+CTI). The experiment was performed 3 times and data from one representative experiment is shown. FU = fluorescence units, TF = tissue factor, CTI = corn trypsin inhibitor, eryMPs = erythrocyte-derived microparticles.

**Figure 3:**
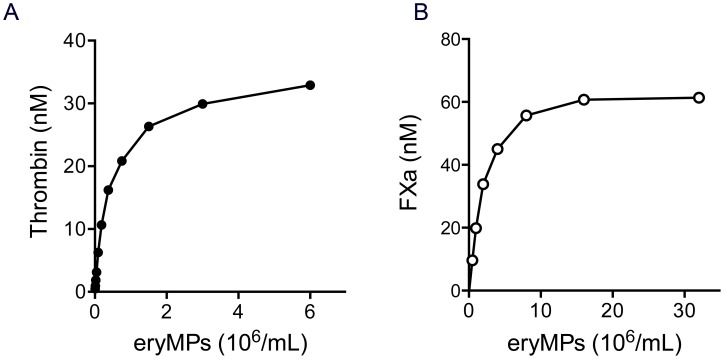
Erythrocyte-derived microparticles supporting PTase and Xase complexes. A) To 50 µL reaction mix (EryMPs, FXa and CaCl_2_), 50 µL FVa and 150 µL prothrombin were added and incubated at 37°C. The reaction was stopped after 2 minutes by dilution in EDTA-containing stop buffer. The thrombin formed was measured kinetically with chromogenic substrate (S-2238). Human thrombin of known concentration was used to obtain a calibration curve for the conversion of the substrate. Final concentrations: 0.5 µM prothrombin, 5 nM hFXa, 40 pM FVa, 3 mM CaCl_2_ and 0–6×10^6^ eryMPs/mL. B) To 60 µL FVIIIa (370 mU/mL) and FIXa (8.9 nM) 40 µL eryMPs (FC: 0–16×10^6^/mL) and bovine FX (FC: 0.5 µM) were added and incubated for 3 minutes. The reaction was stopped by dilution 1/16 in ice-cold EDTA-containing buffer. Formed FXa, was measured kinetically by conversion of the chromogenic substrate S-2765 (Chromogenix). Known concentrations of FXa were used to obtain a calibration curve for the conversion of the substrate.

The MPs were efficient in supporting reactions of blood coagulation as tested by thrombin generation assay (TGA), Xase and PTase reactions. In agreement with results on record, the eryMPs activated the intrinsic pathway, as demonstrated by thrombin generated in the absence of added TF and CTI (Figure. 2A). In the presence of 3×10^6^/ml particles, thrombin peaked after a lag phase of approximately 8 minutes. The addition of CTI to the plasma (50 µg/mL) delayed the thrombin generation, the lag phase reaching around 20 minutes (not shown). The small amount of thrombin that was generated in the absence of TF and CTI despite lack of added MPs was presumably due to activation of the intrinsic pathway by contaminating membranes (e.g. microparticles) in the plasma because no thrombin generation was detected when CTI was included (not shown). The extrinsic pathway was activated by the addition of 0.7 pM TF, the plasma containing CTI (50 µg/mL) to inhibit the intrinsic pathway. At 3×10^6^/mL particles, the lag phase was around 4 minutes long and thrombin peaked at 8 minutes (Figure 2B). The TGA results suggested that the eryMP could provide the appropriate surface to assemble the procoagulant Xase and PTase complexes. This was confirmed in Xase and PTase model systems where the addition of increasing concentrations of MPs resulted in increased formation of FXa and thrombin, maximum reaction rates being reached at MP concentrations per ml of approximately 10×10^6^, and 3×10^6^, respectively (Figure 3).

**Figure 4:**
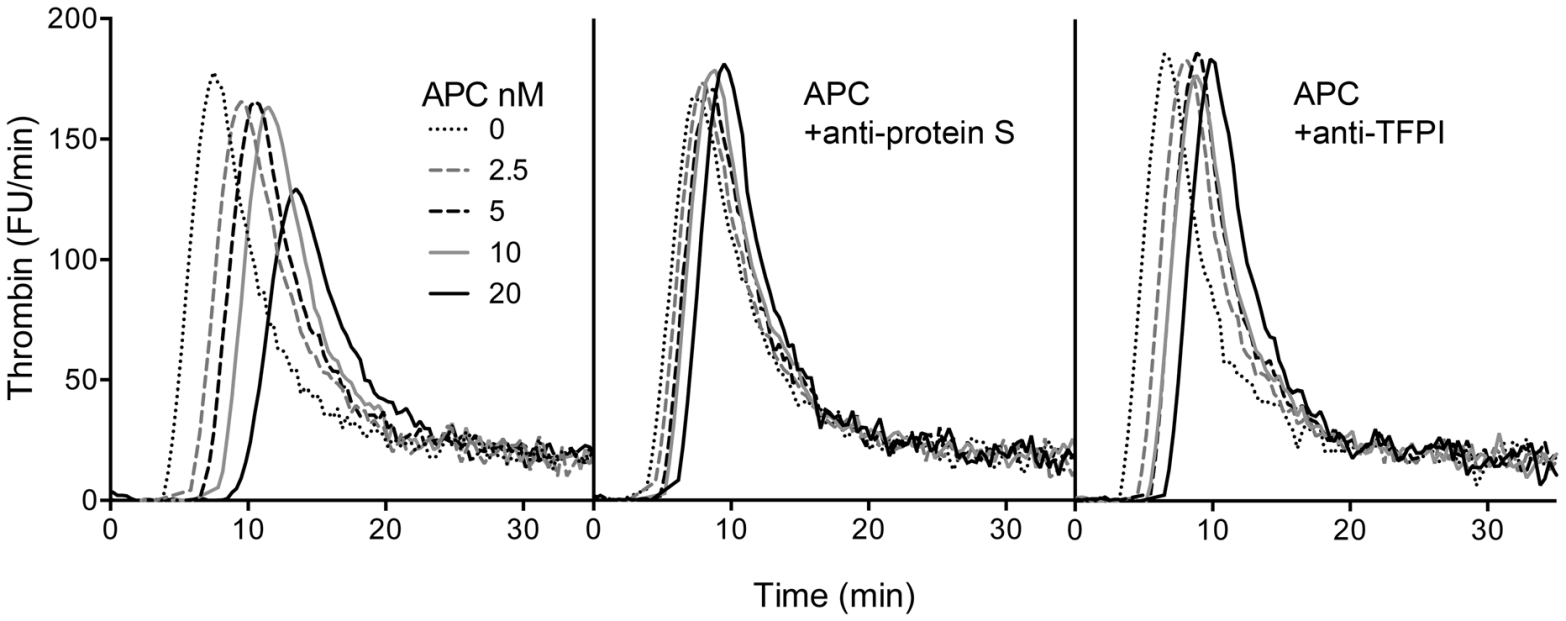
Protein S-dependence of APC-mediated inhibition of thrombin generation on microparticles. 80 µL (diluted ½) platelet poor plasma, supplemented with 50 µg/mL CTI, was mixed with eryMPs (30 µL) ± APC and TF. Thrombin generation was initiated by addition of 20 µL CaCl_2_ solution containing the thrombin substrate Z-Gly-Gly-Arg-AMC. The accumulated fluorescence was monitored and presented as the first derivative representing thrombin activity. Left panel: Thrombin generation in presence of 0–20 nM APC; middle panel: 0–20 nM APC+anti-protein S (monoclonal MK21); right panel: 0–20 nM APC+anti-TFPI (polyclonal). Final concentrations: 0.7 pM TF, 3×10^6^ eryMPs/mL, 0–20 nM APC, 300 µM Z-Gly-Gly-Arg-AMC, 16.7 mM CaCl_2_, 50 µg/mL CTI (in plasma) and 100 µg/mL anti-protein S or anti-TFPI (in diluted plasma). Data are from one representative experiment. FU = fluorescence units. APC = activated protein C, anti-protein S = monoclonal antibody against protein S, anti-TFPI = polyclonal antibody against TFPI.

The addition of increasing concentrations of APC to the TF-initiated TGA resulted in a dose-dependent delay in thrombin generation with prolongation of the lag phase (Figure 4). The anticoagulant effect of APC was dependent on protein S as demonstrated by the addition an anti-protein S monoclonal antibody, which inhibited the anticoagulant effect of APC. Inclusion of a polyclonal antibody against TFPI attenuated the effect of APC, which was mainly observed as shorter lag phase at high concentration of APC. In absence of added APC, the lag phase was shortened by less than one minute upon addition of anti-TFPI. Also in absence of added TF and CTI, i.e. when coagulation was initiated via the intrinsic pathway, the addition of APC resulted in increased lag-time. However, due to great variability between different donors it was difficult to draw valid conclusions on intrinsic pathway activated TGA and anti-coagulant effects of APC (data not shown).

### Erythrocyte microparticles support APC-mediated FVa and FVIIIa degradations

**Figure 5:**
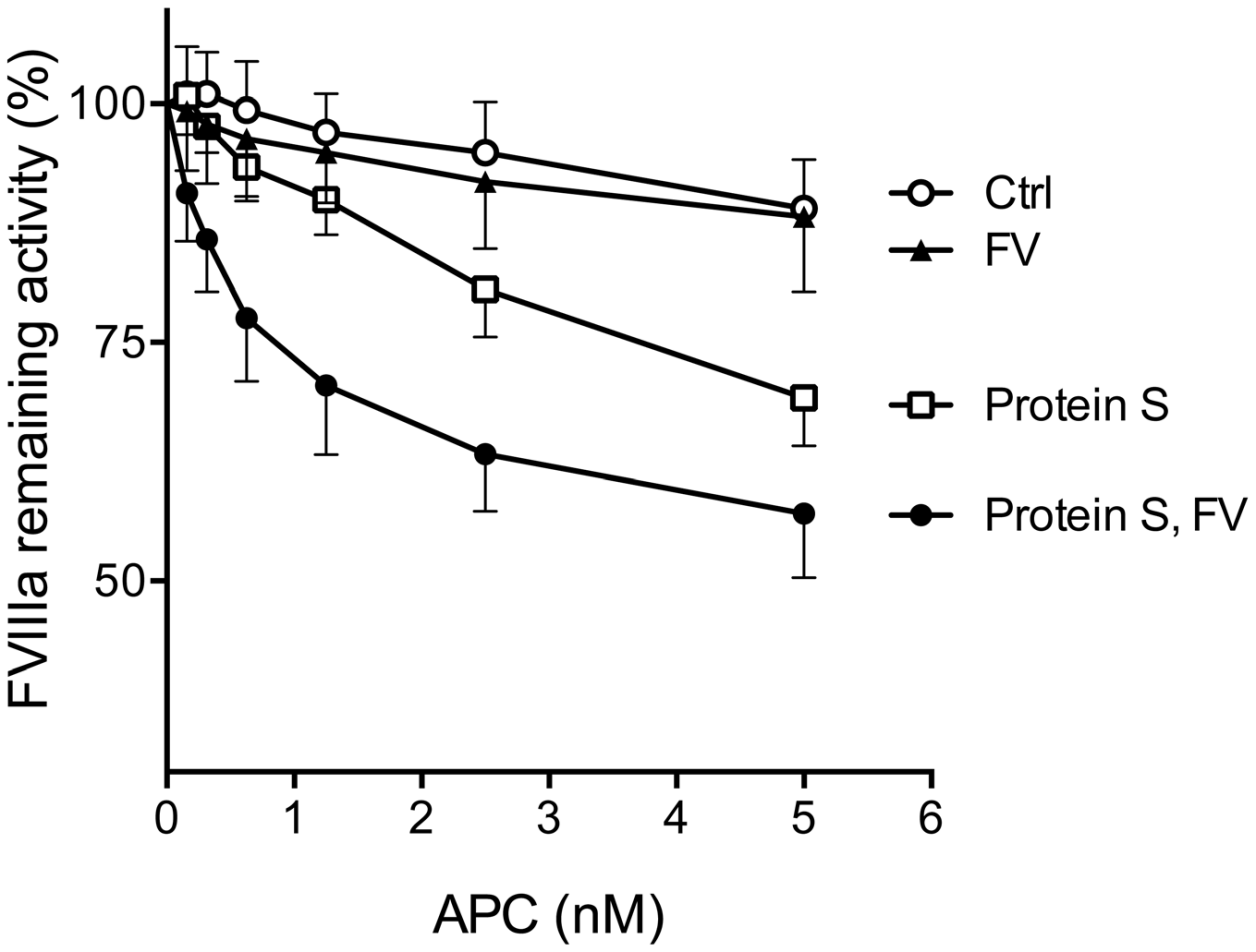
Activated protein C-mediated FVIIIa-degradation on erythrocyte-derived microparticles stimulated by FV and protein S. EryMPs (16×10^6^/mL) were incubated with FVIIIa (212 mU/mL), FIXa (5 nM), APC (0–5 nM) with or without protein S (33 nM) and/or FV (2 nM) at 37°C for 2.5 minutes. FX was added (to 0.5 µM) and after 3 minutes incubation, the activity of formed FXa was measured by conversion of a synthetic colorimetric substrate. Data presented as mean±SD, N = 3, APC = activated protein C, PS = protein S, FV = factor V.

To elucidate whether the Xase complex could be regulated on the surface of eryMPs, the eryMPs with assembled Xase complexes were incubated with increasing concentrations of APC in the presence or absence of the APC-cofactors protein S and FV (Figure 5). APC alone did not affect the Xase activity demonstrating the importance of APC cofactors in the reaction. In the presence of protein S, APC was able to inhibit the Xase, whereas the addition of FV together with APC yielded no inhibition of Xase activity. The combined addition of protein S and FV resulted in a synergistic APC-cofactor function with approximately 5-fold less APC required to yield the same effect as when APC was added in the presence of protein S as only cofactor.

**Figure 6:**
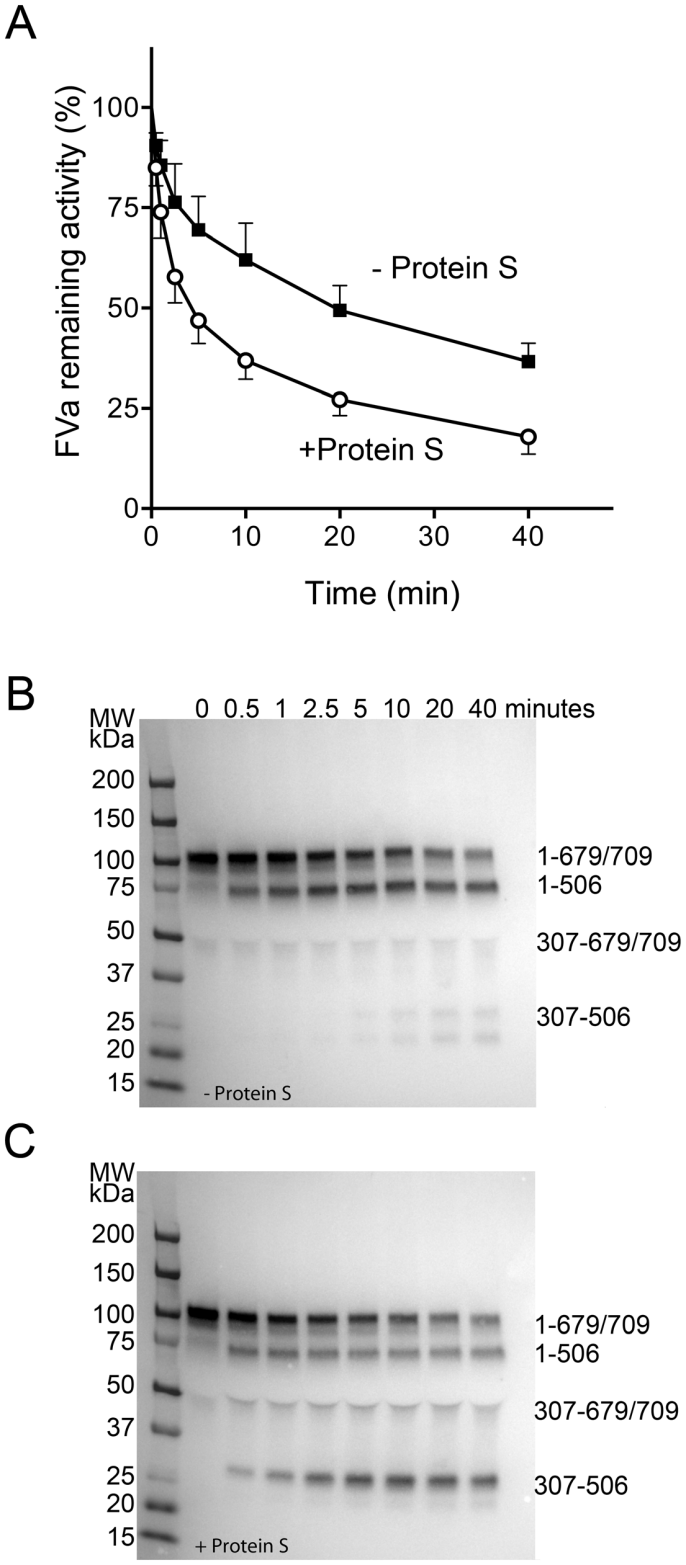
Stimulation by protein S of APC-mediated FVa degradation on erythrocyte-derived microparticles. APC (60 pM) was incubated with FVa (0.8 nM) in presence of 6×10^6^/mL eryMPs with or without 100 nM protein S. A) At intervals, aliquots were drawn and diluted 1/10 in ice cold buffer to stop the reaction and remaining FVa activity analyzed after additional 1/5 dilution in a PTase reaction containing prothrombin (0.5 µM), FXa (5 nM) and extruded phospholipids (PC/PS 90/10 (50 µM)). Data are presented as FVa remaining activity, mean±SD (error bars), N = 3). B, C) Affinity-purified FVa was subjected to APC-mediated degradation in presence (C) or absence (B) or protein S as in A and at intervals, the FVa-degradation was stopped by dilution in an equal volume denaturing, reducing sample buffer and the FV-fragments were separated on a 4–15% SDS-PAGE gel and transferred to a blotting membrane. FVa fragments were detected with anti-FV (AHF-5146 with epitope between 307–506) and visualized with enhanced chemiluminescence. The peptide-composition of the FVa fragments is denoted to the right.

Degradation of FVa by APC in presence or absence of protein S was studied in the FVa degradation assay, the remaining FVa activity being measured with a PTase assay and the fragmentation pattern followed on western blotting (Figure 6). In the absence of protein S, APC (60 pM) efficiently cleaves the Arg506 site, generating a 75 kDa fragment corresponding to residues 1–506. The FVa that is cleaved at Arg506 has remaining activity and at the end of the reaction, 35±5% FVa activity remained. On the Western blotting, a weak intact heavy chain band was also observed at this time point. The loss of FVa activity was almost 3 times faster in presence of protein S, the remaining activity at 40 minutes being 16±3%. The cofactor function of protein S is predominantly efficient for the cleavage at Arg306, which is evidenced from the western blotting where a strong ≈30 kDa band corresponding to the 307–506 fragment is seen. Kinetic rate constants were calculated; k_506_ was 2.3×10^8^ M^−1^s^−1^ and 1.7×10^8^ M^−1^s^−1^ without and with protein S, respectively. The rate constant for the protein S dependent Arg306-cleavage was affected by addition of protein S, increasing from 5.2×10^6^ M^−1^s^−1^ to 7.8×10^6^ M^−1^s^−1^.

## Discussion

Increased concentrations of circulating eryMPs can be found in patients with diseases affecting red blood cells (i.e. sickle cell disease, PNH, malaria, β-thalassemia) [15], [16], [33], [34]. These diseases are prothrombotic in nature, having signs of activation of blood coagulation, and are often complicated by localized or generalized thrombotic disease. The increased concentration of circulating eryMPs presumably contributes to the procoagulant phenotype due to the exposure of negatively charged phospholipids that can assemble procoagulant reactions. Another source of eryMPs is transfusion of ageing erythrocyte concentrate because upon storage, erythrocytes constitutively shed MPs. Hence; all patients receiving blood transfusions are at risk for having increased concentration of circulating eryMPs. Indeed, a link between blood unit storage time (i.e. concentration of eryMPs) and transfusion complications has been described [35], [36].

Negatively charged phospholipids are not only important for the assembly of procoagulant reactions but are also required for the activity of the anticoagulant protein C system. It is therefore likely that the negatively charged phospholipids that are exposed on eryMPs not only support procoagulant pathways but also the anticoagulant protein C system. In the present study, both pro- and anticoagulant effects of both A23187-induced and outdated eryMPs were investigated. Like naturally occurring circulating eryMPs, both these MPs expose the negatively charged phospholipid phosphatidylserine, which is essential for their ability to support reactions involved in blood coagulation and its regulation. We have primarily used the A23187-induced eryMPs but in control experiments shown that outdated eryMPs behave qualitatively similar. Our presented results demonstrate that protein S binds to the eryMPs and support that anticoagulant activity of APC in thrombin generation assays and in purified systems investigating FVa- and FVIIIa- degradations.

Activation of cells with calcium ionophore A23187 leads to increase of intracellular calcium, which is a common cell response upon exposure to natural agonists. The increase in intracellular calcium causes dislocation of membrane phospholipids, leading to exposure of internal phospholipids in the outer leaflet. Membrane anchoring to intracellular cytoskeleton is disrupted and microvesicles can be formed. Under physiological conditions, natural agonists activate cells by different pathways, which leads to elevation of intracellular calcium. The outcome is hence similar to the use of artificial A23187 as agonist, and several experimental studies have used A23187 to investigate erythrocyte-derived MPs [17], [37]. Furthermore, in a study by Salzer et. al, A23187-generated eryMPs were demonstrated to have properties similar to the natural occurring MPs isolated form outdated erythrocyte concentrates (datEryMPs) [38]. We have performed supplemental experiments, using datEryMPs, isolated by the method described by Rubin et. al [27] and found that the eryMPs of the two different sources had equivalent characteristics in terms of phosphatidylserine exposure, pro- and anticoagulant properties.

The A23187-generated eryMPs used in this study were characterized by flow cytometry. In line with several other studies, the eryMPs did expose phosphatidylserine in the outer leaflet as detected through binding of fluorescently labeled lactadherin [28]. The exposure of phosphatidylserine also provided a suitable membrane surface for binding of added protein S, as detected by flow cytometry. Flow cytometry was also used to measure the concentration of MPs in the preparations. Erythrocyte-derived MPs have been reported to be smaller than other MPs as determined by electron microscopy (down to 0.15 µm) [39] and due to the resolution limitations in the flow cytometer, the concentrations used in our assays were probably underestimated. A possible way to normalize MP-concentrations is to measure the phosphatidylserine concentration. However, this approach would not contribute to clarify the actual particle-number, which is of interest when comparing with MP-concentrations in vivo.

Studies of patients with sickle cell anemia and β-thalassemia report increased thrombin generation in vivo correlated to presence of red blood cells or MPs exposing negatively charged phospholipids [16]. Moreover, decreased levels of classical intrinsic proteins like FXII, prekallikrein, high molecular weight kininogen and FXI have been reported in patients with sickle cell disease [40]. This goes in line with the observation that eryMPs activate the intrinsic pathway in a FXII-dependent manner. However the effect was not simply due to presence of negatively charged membrane, since phospholipid vesicles containing phosphatidylserine did not trigger coagulation [17]. Others have ruled out presence of polyphosphates as possible activator in eryMP preparations [6]. Thus, it is presently unclear what is initiating contact activation by eryMPs.

The anticoagulant efficiency of APC on eryMPs depended on the presence of its cofactor protein S. Thus, in the plasma-based TGA system, the addition of a neutralizing antibody against protein S decreased the anticoagulant effect of APC. In the FVa-degradation, protein S stimulated the Arg306 cleavage, increasing the generation of the 307–506 fragment. However, the calculated cleavage rate constants showed only a moderate protein S effect, protein S stimulating the Arg306-cleavage 1.5 times. This is a smaller protein S effect than reported on the surface of phospholipids [41]–[43]. By using the pseudo first order equation to estimate cleavage rates of normal FVa, there is a possibility that the protein S effect is underestimated due to the absence of a clear distinction between the two phases of degradation, the fast 506-cleavage and the slower 306-cleavage. To allow analysis of the protein S enhancement of k_306_, degradation of recombinant FV506Q/679Q would be required. The purpose of the calculated rates was however to compare the efficiency of the APC-system on eryMPs, with present data using phospholipid vesicles as membrane source. In the FVIIIa-degradation, full APC effect required the combined presence of both protein S and FV.

Protein S has been reported to have APC-independent anticoagulant functions. Apart from being a cofactor to APC, it interacts with TFPIα and supports the action of TFPIα in the regulation of the TF-initiated coagulation [44]–[48]. However, we could not detect any APC-independent effect of protein S on the surface of eryMPs in the TGA. Inclusion of an antibody against protein S in absence of APC did not affect the thrombin generation curve as shown before using both phospholipids [44] and platelet-derived MPs [20]. Platelets contain TFPIα, which is released upon activation [49], [50]. Also FV resides within platelets [51], [52] and has been reported to bind TFPIα [53]. It is plausible that platelet-derived MPs bind TFPIα, either direct, or via FV or protein S on their surface. There are no indications that eryMPs contain TFPI, which could explain the discrepancy in comparison with platelet-derived MPs but not with phospholipids. When we included an antibody against TFPI in the thrombin generation assay with eryMPs, a shortening of the lag phase both in absence and in presence of APC was observed. The shortening of lag phase seen in presence of APC+anti-TFPI as compared to APC alone could be due to increased TF activity due to the lack of TFPI-mediated regulation. The resulting increased procoagulant load would in turn make APC seemingly less efficient. However, it cannot be ruled out that there are unknown cross-reactions between the two regulatory systems, TFPI and APC, an interesting topic for additional studies.

Hypercoagulable states associated with increased levels of eryMPs, such as sickle cell disease and β-thalassemia, are also associated with low levels of protein C and free protein S [14], [54]. This could be explained by consumption due to coagulation activation. However, the binding of protein S to MPs and red blood cells exposing phosphatidylserine may enhance the elimination of protein S from circulation [18].

In conclusion, the role of eryMPs in the pathophysiology of different diseases is poorly understood. This study contributes knowledge on the APC-mediated regulation of thrombin formation on eryMPs, which may be physiologically important for suppression of the procoagulant effects of eryMPs.

## Supporting Information

Figure S1 **Exposure of phosphatidylserine on the surface of outdated erythrocyte-derived MPs.** The outdated eryMP were analyzed essentially as described in the manuscript with the addition that also annexin V staining was performed.
(TIF)

Figure S2 **datEryMPs support formation of FXa and thrombin in the Xase and prothrombinase reactions.** A) To 50 µL reaction mix (datEryMPs, FXa and CaCl_2_), 50 µL FVa and 150 µL prothrombin were added and incubated at 37°C. The reaction was stopped after 2 minutes by dilution in EDTA-containing stop buffer. The thrombin formed was measured kinetically with chromogenic substrate (S-2238). Final concentrations: 0.5 µM prothrombin, 5 nM hFXa, 40 pM FVa, 3 mM CaCl_2_ and 0–50×10^6^ eryMPs/mL. B) To 60 µL FVIIIa (370 mU/mL) and FIXa (8.9 nM) 40 µL datEryMPs (FC: 0–50×10^6^/mL) and bovine FX (FC: 0.5 µM) were added and incubated for 3 minutes. The reaction was stopped by dilution in ice-cold EDTA-containing buffer. Formed FXa, was measured kinetically by conversion of the chromogenic substrate S-2765 (Chromogenix).
(TIF)

Figure S3 **datEryMPs supporting the FVa-degradation by APC.** APC (60 pM) was incubated with FVa (0.8 nM) in presence of 50×10^6^/mL eryMPs with (○) or without (•) 100 nM protein S. At intervals, aliquots were drawn and diluted 1/10 in ice cold buffer to stop the reaction and remaining FVa activity was analyzed after additional 1/5 dilution in a PTase reaction containing prothrombin (0.5 µM), FXa (5 nM) and extruded phospholipids (PC/PS 90/10 (50 µM)). Data are presented as FVa remaining activity.
(TIF)

Figure S4 **datEryMPs supporting APC-mediated FVIIIa degradation.** EryMPs (25×10^6^/mL) were incubated with FVIIIa (212 mU/mL), FIXa (5 nM), APC (0–5 nM), protein S (33 nM) and/or FV (2 nM) at 37°C for 2.5 minutes. FX was added (to 0.5 µM) and after 3 minutes incubation, the activity of formed FXa was measured by conversion of a synthetic colorimetric substrate. Data are presented as FVIIIa remaining activity. The data from the A23187-derived eryMPs in grey are those from the manuscript and are shown for comparison.
(TIF)

Figure S5 **Dose dependent increment of thrombin generation in plasma in presence of increasing concentrations of datEryMPs.** Platelet poor plasma supplemented with 50 µg/mL CTI was diluted ½ and 80 µL was added to a mix of datEryMPs and TF (30 µL). Thrombin generation was initiated by addition of 20 µL CaCl_2_ solution containing the thrombin substrate Z-Gly-Gly-Arg-AMC. The accumulated fluorescence was monitored and presented as the first derivative representing thrombin activity. Concentrations in the assay were: 0–12×10^6^ datEryMPs/mL, 0.7 pM TF, 300 µM Z-Gly-Gly-Arg-AMC, 16.7 mM CaCl_2_. FU = fluorescence units, TF = tissue factor, CTI = corn trypsin inhibitor, datEryMPs = microparticles isolated from outdated erythrocyte concentrates.
(TIF)

Figure S6 **APC-mediated reduction of thrombin generation in plasma supported by protein S.** 80 µL (diluted ½) platelet poor plasma, supplemented with CTI, was mixed with eryMPs (30 µL) ± APC and TF. Thrombin generation was initiated by addition of 20 µL CaCl_2_ solution containing the thrombin substrate Z-Gly-Gly-Arg-AMC. The accumulated fluorescence was monitored and presented as the first derivative representing thrombin activity. Left panel: Thrombin generation in presence of 0–20 nM APC; middle panel: 0–20 nM APC+anti-protein S (monoclonal MK21); right panel: 0–20 nM APC+anti-TFPI (polyclonal). Final concentrations: 0.7 pM TF, 3×10^6^ eryMPs/mL, 0–20 nM APC, 300 µM Z-Gly-Gly-Arg-AMC, 16.7 mM CaCl_2_, 50 µg/mL CTI (in plasma) and 100 µg/mL anti-protein S or anti-TFPI (in diluted plasma). Data from one representative experiment. FU = fluorescence units. APC = activated protein C, anti-protein S = monoclonal antibody against protein S, anti-TFPI = polyclonal antibody against TFPI.
(TIF)

Text S1 **Result of supplemental experiments performed on eryMPs isolated from outdated erythrocyte concentrates.**
(DOC)
