# Supplementary figures and images for: Erythrocyte-Derived Microparticles Supporting Activated Protein C-Mediated Regulation of Blood Coagulation
Source: PLoS One. 2014 Aug 19;9(8):e104200. doi: 10.1371/journal.pone.0104200 (PMC4138094; doi:10.1371/journal.pone.0104200)

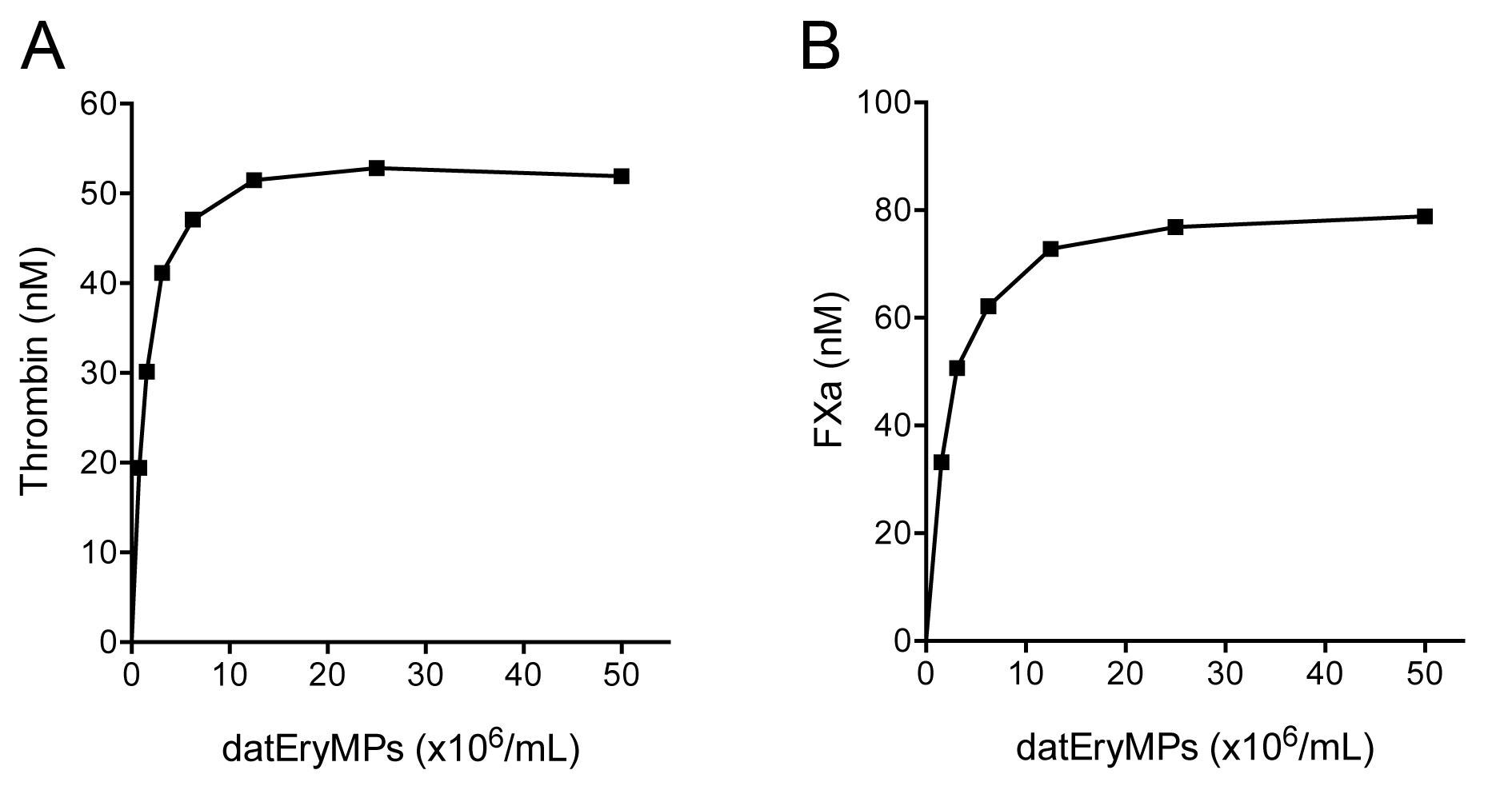

Supplement: Figure S2 — datEryMPs support formation of FXa and thrombin in the Xase and prothrombinase reactions. A) To 50 µL reaction mix (datEryMPs, FXa and CaCl2), 50 µL FVa and 150 µL prothrombin were added and incubated at 37°C. The reaction was stopped after 2 minutes by dilution in EDTA-containing stop buffer. The thrombin formed was measured kinetically with chromogenic substrate (S-2238). Final concentrations: 0.5 µM prothrombin, 5 nM hFXa, 40 pM FVa, 3 mM CaCl2 and 0–50×106 eryMPs/mL. B) To 60 µL FVIIIa (370 mU/mL) and FIXa (8.9 nM) 40 µL datEryMPs (FC: 0–50×106/mL) and bovine FX (FC: 0.5 µM) were added and incubated for 3 minutes. The reaction was stopped by dilution in ice-cold EDTA-containing buffer. Formed FXa, was measured kinetically by conversion of the chromogenic substrate S-2765 (Chromogenix). (TIF) [file pone.0104200.s002.tif]

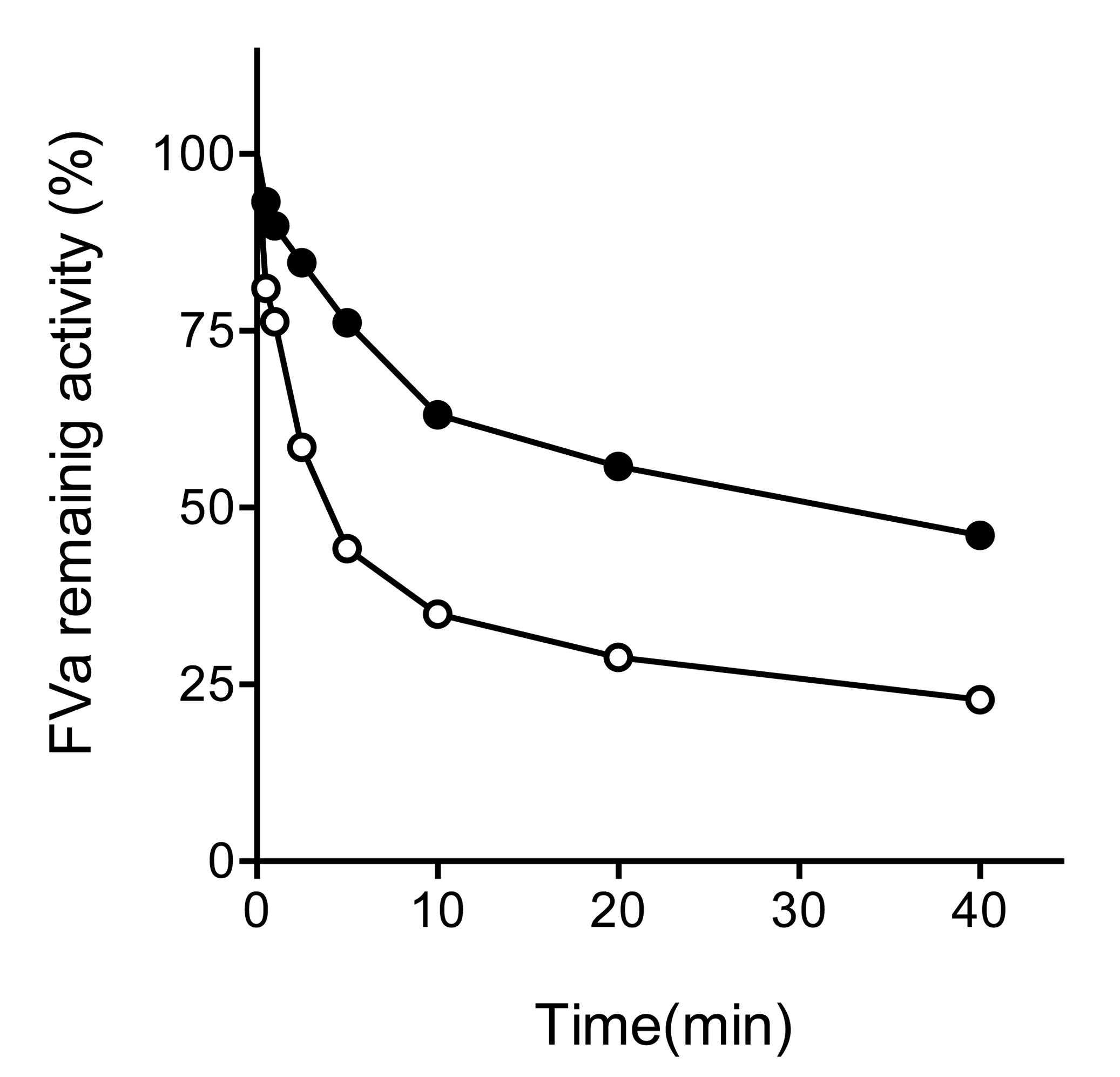

Supplement: Figure S3 — datEryMPs supporting the FVa-degradation by APC. APC (60 pM) was incubated with FVa (0.8 nM) in presence of 50×106/mL eryMPs with (○) or without (•) 100 nM protein S. At intervals, aliquots were drawn and diluted 1/10 in ice cold buffer to stop the reaction and remaining FVa activity was analyzed after additional 1/5 dilution in a PTase reaction containing prothrombin (0.5 µM), FXa (5 nM) and extruded phospholipids (PC/PS 90/10 (50 µM)). Data are presented as FVa remaining activity. (TIF) [file pone.0104200.s003.tif]

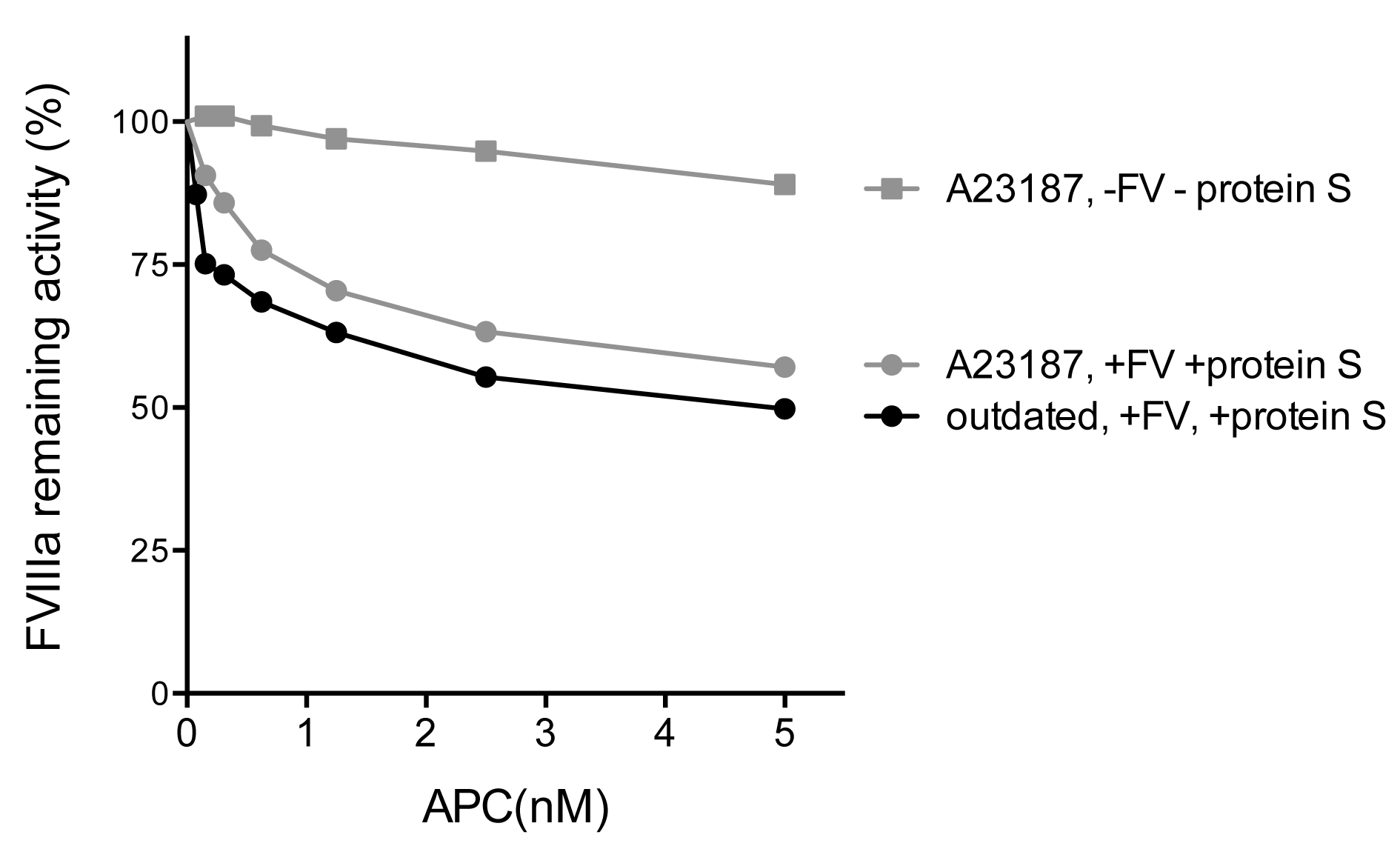

Supplement: Figure S4 — datEryMPs supporting APC-mediated FVIIIa degradation. EryMPs (25×106/mL) were incubated with FVIIIa (212 mU/mL), FIXa (5 nM), APC (0–5 nM), protein S (33 nM) and/or FV (2 nM) at 37°C for 2.5 minutes. FX was added (to 0.5 µM) and after 3 minutes incubation, the activity of formed FXa was measured by conversion of a synthetic colorimetric substrate. Data are presented as FVIIIa remaining activity. The data from the A23187-derived eryMPs in grey are those from the manuscript and are shown for comparison. (TIF) [file pone.0104200.s004.tif]

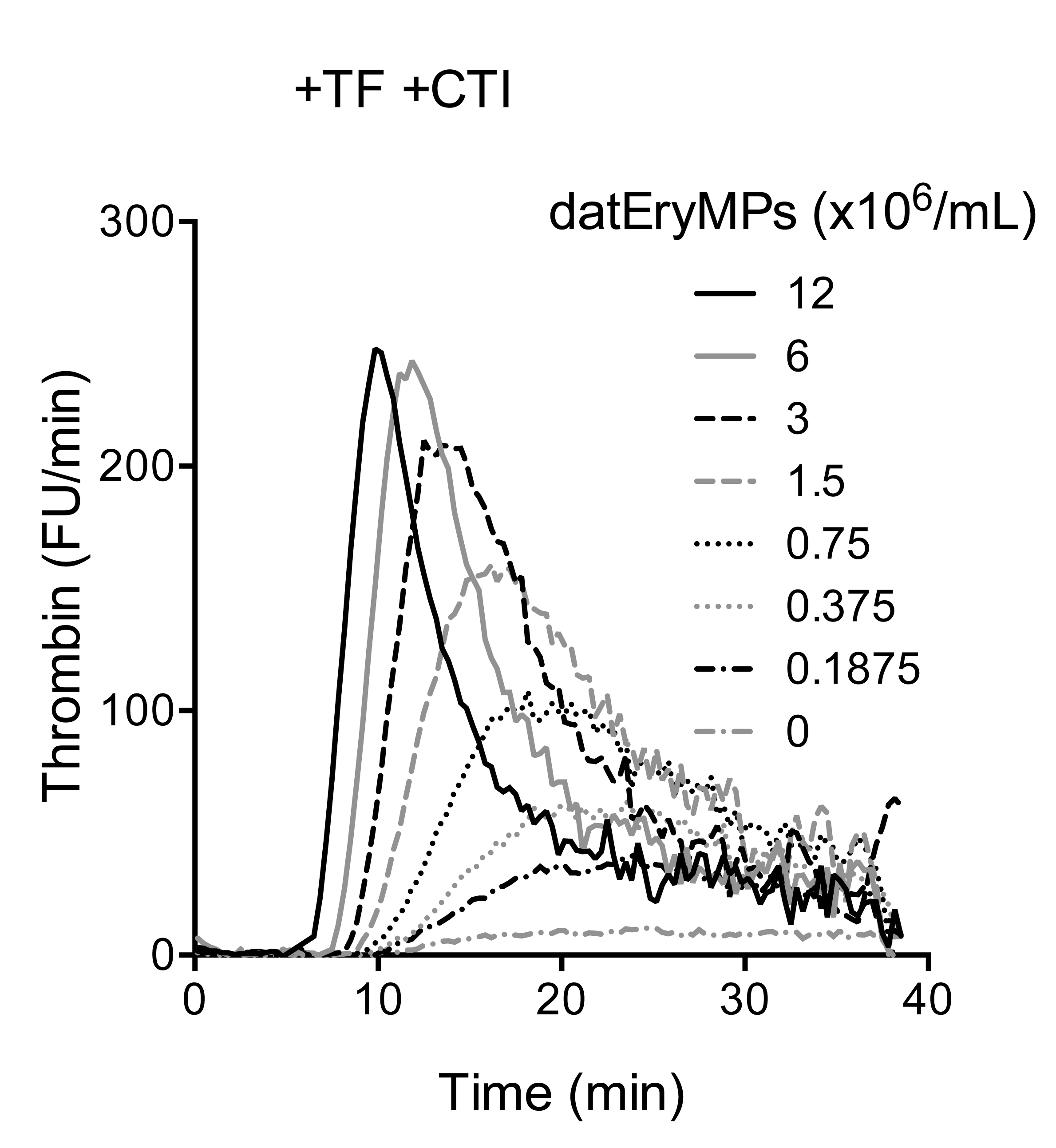

Supplement: Figure S5 — Dose dependent increment of thrombin generation in plasma in presence of increasing concentrations of datEryMPs. Platelet poor plasma supplemented with 50 µg/mL CTI was diluted ½ and 80 µL was added to a mix of datEryMPs and TF (30 µL). Thrombin generation was initiated by addition of 20 µL CaCl2 solution containing the thrombin substrate Z-Gly-Gly-Arg-AMC. The accumulated fluorescence was monitored and presented as the first derivative representing thrombin activity. Concentrations in the assay were: 0–12×106 datEryMPs/mL, 0.7 pM TF, 300 µM Z-Gly-Gly-Arg-AMC, 16.7 mM CaCl2. FU = fluorescence units, TF = tissue factor, CTI = corn trypsin inhibitor, datEryMPs = microparticles isolated from outdated erythrocyte concentrates. (TIF) [file pone.0104200.s005.tif]

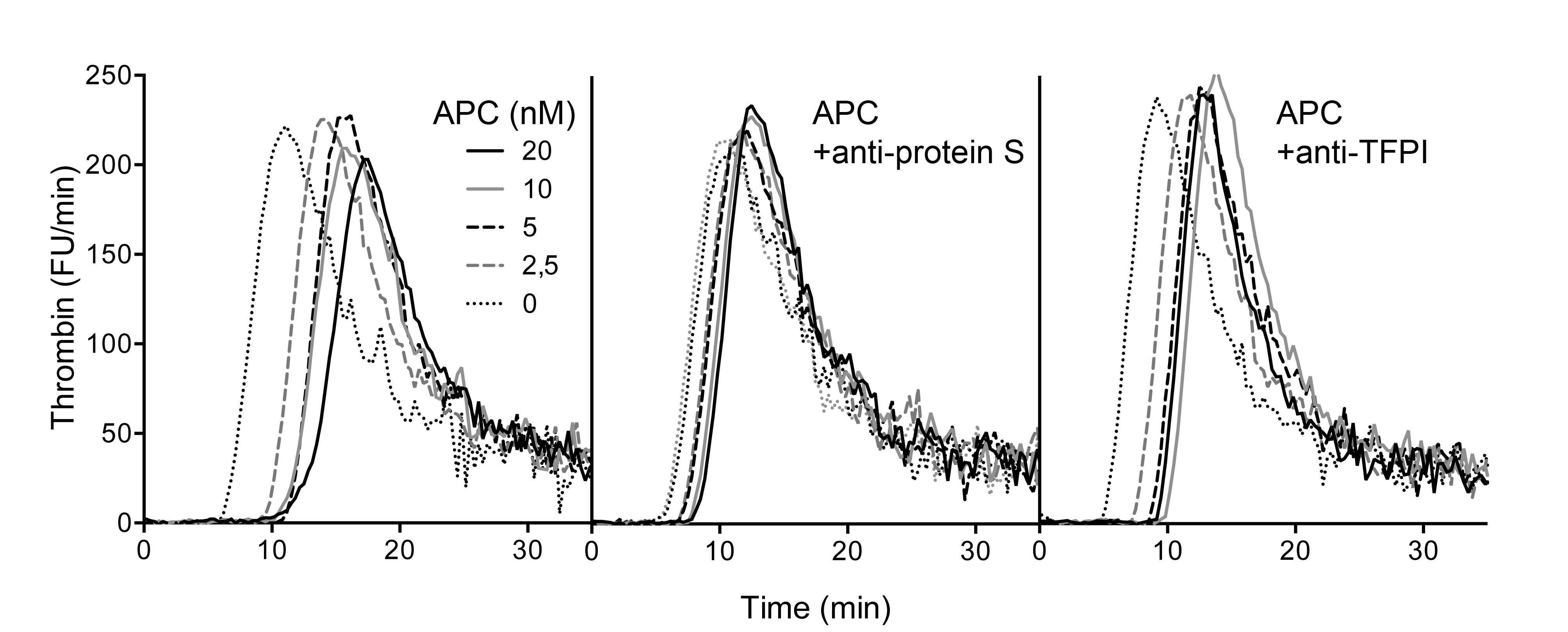

Supplement: Figure S6 — APC-mediated reduction of thrombin generation in plasma supported by protein S. 80 µL (diluted ½) platelet poor plasma, supplemented with CTI, was mixed with eryMPs (30 µL) ± APC and TF. Thrombin generation was initiated by addition of 20 µL CaCl2 solution containing the thrombin substrate Z-Gly-Gly-Arg-AMC. The accumulated fluorescence was monitored and presented as the first derivative representing thrombin activity. Left panel: Thrombin generation in presence of 0–20 nM APC; middle panel: 0–20 nM APC+anti-protein S (monoclonal MK21); right panel: 0–20 nM APC+anti-TFPI (polyclonal). Final concentrations: 0.7 pM TF, 3×10^6^ eryMPs/mL, 0–20 nM APC, 300 µM Z-Gly-Gly-Arg-AMC, 16.7 mM CaCl2, 50 µg/mL CTI (in plasma) and 100 µg/mL anti-protein S or anti-TFPI (in diluted plasma). Data from one representative experiment. FU = fluorescence units. APC = activated protein C, anti-protein S = monoclonal antibody against protein S, anti-TFPI = polyclonal antibody against TFPI. (TIF) [file pone.0104200.s006.tif]
