# Supplementary material for: Erythrocyte-Derived Microparticles Supporting Activated Protein C-Mediated Regulation of Blood Coagulation
Source: PLoS One. 2014 Aug 19;9(8):e104200. doi: 10.1371/journal.pone.0104200 (PMC4138094; doi:10.1371/journal.pone.0104200)
Supplement: Text S1 — Result of supplemental experiments performed on eryMPs isolated from outdated erythrocyte concentrates. (DOC) [file pone.0104200.s007.doc]

**Supplemental results**

In the present study “Erythrocyte-derived microparticles supporting activated protein C-mediated regulation of blood coagulation” artificial microparticles released upon stimulation with the ionophore A23187 were used to study the function of APC-mediated regulation of coagulation. Translation of the results to natural occurring eryMPs in vivo require that the characteristics of the A23187-derived MPs also are valid for these MPs. A previous publication by Salzer et. al demonstrated similar characteristics of A23187eryMPs and eryMPs from outdated erythrocyte concentrates (datEryMPs) regarding MPs size, phosphatidylserine exposure and ability to support thrombin generation in plasma [1]. To verify that the results and conclusions obtained by using A23187eryMPs in the present study also can be assigned to natural occurring erythrocyte derived MPs, such MPs were isolated from outdated erythrocyte concentrates, and additional experiments were performed.

Natural microparticles (datEryMPs) were isolated from outdated (storage time >45 days) erythrocyte concentrates according to the method described by Rubin et. al [2]. The obtained datEryMPs were tested for phosphatidylserine exposure, ability to support thrombin generation in plasma and formation of thrombin and FXa in assays using purified components. Moreover, their ability to support the functions of the activated protein C (APC)-system was investigated.

Flow cytometry analysis of the datEryMPs showed that phosphatidylserine was exposed, as detected with both lactadherin [3] and annexin V (Fig. S1). The negatively charged surface is a prerequisite for the function of both pro- and anticoagulant processes. In purified systems, the ability of the datEryMPs to support formation of FXa and thrombin in the Xase and prothrombinase complexes was investigated (Fig. S2). Both reactions were supported by datEryMPs, however about the double concentration of MPs was needed to reach maximum, as compared with the A23187-derived MPs. One possible explanation could be that the isolated datEryMPs, although being phosphatidylserine positive, exposed lower surface concentration of phosphatidylserine. Moreover, the protocol for isolation of A23187-derived MPs included an over night ultracentrifugation to increase the yield of MPs, which possibly results in isolation of smaller particles that may not be detected by flow cytometry. It is therefore plausible that the concentration of A23187-derived MPs was underestimated. In order to correct for this, the concentration of datEryMPs was increased in assays where the APC-function in FVa (Fig. S3) and FVIIIa-degradation (Fig. S4) was evaluated. The datEryMPs supported FVa-degradation to the same extent as A23187-derived eryMPs, protein S stimulating the reaction. The remaining FVa-activity at 40 minutes in presence of protein S was 16% using A23187eryMPs and 22% with datEryMPs. Also the FVIIIa-degradation in presence of FV and protein S was equally efficient as using A23187eryMPs, with a remaining activity of about 50% at the highest tested APC-concentration.

The datEryMPs were also titrated in the thrombin generation assay (Fig. S5). In presence of tissue factor (TF) and corn trypsin inhibitor (CTI), increasing concentrations of added datEryMPs resulted in increased thrombin generation and shortening of the lag phase. At the highest tested concentration, the lag phase was approximately 8 minutes, which is longer than obtained with the A23187eryMPs. This could however also be explained by either decreased phosphatidylserine exposure or the shorter ultracentrifugation time, as discussed above. Addition of APC in the thrombin generation assay resulted in dose dependent reduction of the lag phase and inclusion of an anti-protein S antibody suppressed the APC-function (Fig. S6). As with the A23187eryMPs, addition of anti-TFPI antibody resulted in shorter lag phase both in absence and presence of APC.

Taken together, erythrocyte-derived MPs isolated from outdated erythrocyte concentrates behave like A23187eryMPs, and the results and conclusions described in “Erythrocyte-derived microparticles supporting activated protein C-mediated regulation of blood coagulation” can therefore be considered valid for natural occurring erythrocyte-derived MPs.

**References**

1. Salzer U, Zhu R, Luten M, Isobe H, Pastushenko V, et al. (2008) Vesicles generated during storage of red cells are rich in the lipid raft marker stomatin. Transfusion 48: 451-462.

2. Rubin O, Crettaz D, Canellini G, Tissot JD, Lion N (2008) Microparticles in stored red blood cells: an approach using flow cytometry and proteomic tools. Vox sanguinis 95: 288-297.

3. Shi J, Shi Y, Waehrens LN, Rasmussen JT, Heegaard CW, et al. (2006) Lactadherin detects early phosphatidylserine exposure on immortalized leukemia cells undergoing programmed cell death. Cytometry Part A : the journal of the International Society for Analytical Cytology 69: 1193-1201.
